# Relation between magnetopause position and reconnection rate under quasi-steady solar wind dynamic pressure

**DOI:** 10.1186/s40623-024-02101-9

**Published:** 2024-12-18

**Authors:** Hyangpyo Kim, Hyunju Kim Connor, Ying Zou, Jaeheung Park, Rumi Nakamura, Kathryn McWilliams

**Affiliations:** 1 Space Research Institute, Austrian Academy of Sciences, Graz, Austria; 2 NASA Goddard Space Flight Center, Greenbelt, MD USA; 3 Johns Hopkins University Applied Physics Laboratory, Laurel, MD USA; 4 Korea Astronomy and Space Science Institute, Daejeon, South Korea; 5 Department of Astronomy and Space Science, Korea University of Science and Technology, Daejeon, South Korea; 6 Department of Physics and Engineering Physics, University of Saskatchewan, Saskatoon, SK Canada

**Keywords:** Magnetopause, Reconnection, Reconnection rate, Open–closed magnetic field boundary, SuperDARN

## Abstract

The lunar environment heliospheric X-ray imager (LEXI) and solar wind–magnetosphere–ionosphere link explorer (SMILE) will observe the magnetopause motion in soft X-rays to understand dayside reconnection modes as a function of solar wind conditions after their respective launches in the near future. To support their successful science mission, we investigate the relationship between the magnetopause position and the dayside reconnection rate by utilizing super dual auroral radar network (SuperDARN) observations and widely used empirical models of magnetopause position (Shue et al. in J Geophys Res 103:17691–17700. 10.1029/98JA01103, 1998 and Lin et al. in J Geophys Res 115:A04207. 10.1029/2009JA014235, 2010). We select three cases when the interplanetary magnetic field rotates during periods of quasi-steady solar wind dynamic pressure. We first estimate the dayside reconnection rate by calculating the electric field along the open–closed magnetic field boundary (OCB) in the OCB moving reference frame. Then, we estimate the magnetopause position near the local noon by inputting NASA OMNI solar wind data into the empirical magnetopause models. The reconnection rate shows anti-correlation with the magnetopause position that it generally increases as the magnetopause located closer to Earth and vice versa. Our result also confirms that the reconnection rate increases as the empirical coupling efficiency between solar wind and the magnetosphere increases.

**Graphical abstract:**

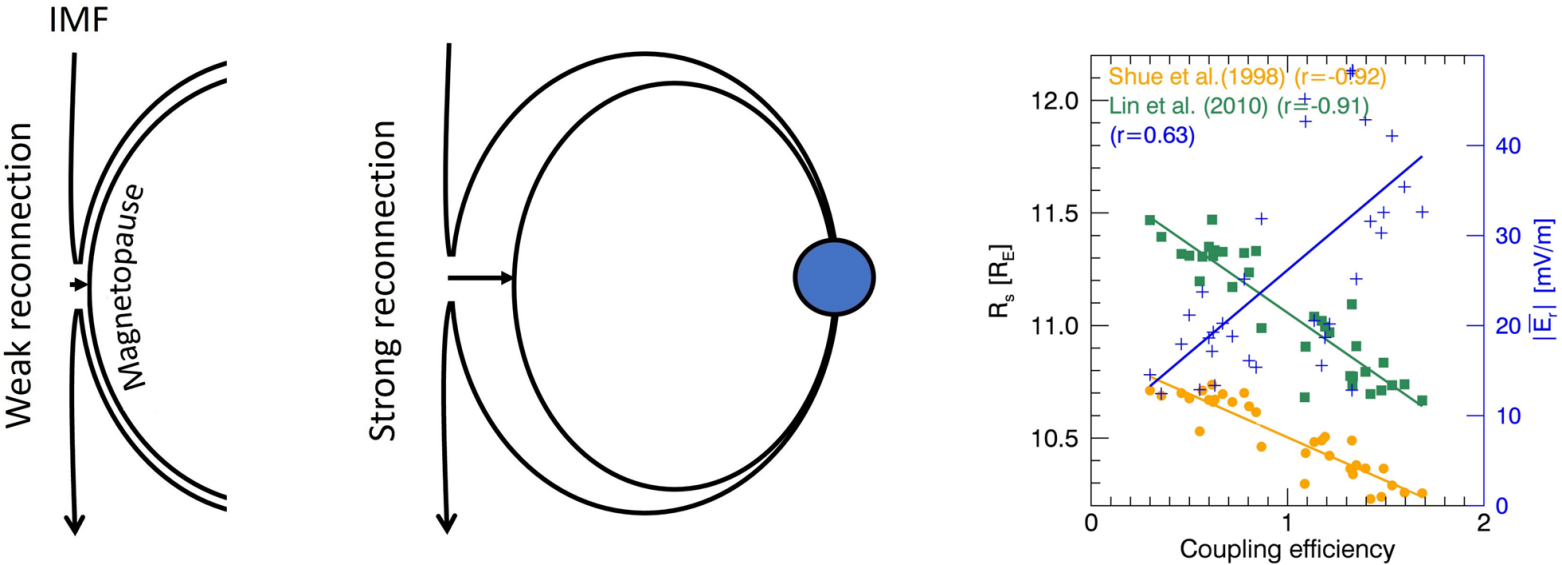

**Supplementary Information:**

The online version contains supplementary material available at 10.1186/s40623-024-02101-9.

## Introduction

The shape and position of the dayside magnetopause undergo constant adjustments, maintaining equilibrium between the magnetosphere and the surrounding solar wind plasma (Chapman and Ferraro 1931; Spreiter et al. 1966). The magnetopause position at the subsolar point is typically known to be 8–13 Earth Radii (${R}_{\text{E}}$) from the Earth’s center, and it shifts with changing solar wind dynamic pressure and north–south components of the Interplanetary Magnetic Field (IMF) (Sibeck et al. 1991). Observations from the Ogo5 satellite by Aubry et al. (1970) demonstrated that north-to-south turning of the IMF causes an inward motion of the magnetopause. This finding was subsequently confirmed by Fairfield (1971), Maezawa (1974), Petrinec et al. (1991), and Tsyganenko and Sibeck (1994), revealing that the magnetopause position is 0.5–1.5 ${R}_{\text{E}}$ closer to the Earth during southward IMF compared to periods of northward IMF, despite identical solar wind dynamic pressures. Therefore, it is practically accepted that the magnetopause is closer to the Earth when affected by a southward IMF. The magnetopause can occasionally approach the geosynchronous orbit during intense geomagnetic storms accompanied by strong solar wind dynamic pressure (e.g., Le et al. 2016; Kim et al. 2024).

The earthward motion of the magnetopause during southward IMF has been attributed to the result of dayside magnetic reconnection (hereafter reconnection). Reconnection between the IMF and the Earth’s magnetic field erodes the magnetopause by creating open field lines at both hemispheres and reduces the closed magnetic flux on the dayside. The open field lines are subsequently carried tailward by the solar wind, creating ionospheric antisunward convection flow. These open field lines are reclosed by tail reconnection and finally return to the dayside with sunward convection flow, restoring the initial magnetospheric configuration. This cyclic process is known as the Dungey cycle (Dungey 1961). The Dungey cycle is generally under equilibrium when the reconnection rates of the dayside and nightside are equal. However, if the reconnection rate in the dayside surpasses that of the nightside, it can lead to magnetopause erosion (e.g., Aubry et al. 1970; Holzer and Salvin 1979; Wing and Sibeck 1997; Mühlbachler et al. 2003; Wiltberger et al. 2003). During reconnection, solar wind particles infiltrate the Earth’s magnetosphere and contribute to associated currents, such the Region 1 field-aligned current and the ring current. This influx results in a pressure imbalance at the magnetopause, driving changes in its configuration (Russell et al. 1974; Hill and Rassbach 1975; Maltsev and Lyatsky 1975; Sibeck et al. 1991; Sibeck 1994).

The reconnection rate quantifies how fast magnetic flux is transferred through the reconnection X line, i.e., the temporal rate of magnetic flux change that undergoes the reconnection process, and indicates how much magnetic energy is released in a certain time. The reconnection rate can be calculated as the electric field measured at either the magnetopause reconnecting X-line (or separatrix) or at the high latitude ionosphere, i.e., open–closed magnetic field line boundary (OCB). Beaujardiere et al. (1991), utilizing data from the Sondrestrom incoherent scatter radar, calculated the reconnection electric fields at the nightside. These fields were found to be less than 15 mV/m during local polar cap expansion and 30 to 40 mV/m during periods of polar cap contraction. Hubert et al. (2006), employing the far ultraviolet imager onboard the imager for magnetopause-to-aurora global exploration (IMAGE) satellite and the super dual auroral radar network (SuperDARN), assessed electric potential as the reconnection rate at both dayside and nightside during substorm intervals. Their findings indicated that the integrated nightside reconnection peaks at the onset of the substorm expansion phase or shortly thereafter, reaching ~ 120 kV before gradually returning to ~ 30 kV. In a simulation study focusing on solar wind pressure enhancements, Connor et al. (2014) demonstrated that strong dynamic pressure intensifies both dayside and nightside reconnection rates. Furthermore, they also showed that during periods of southward IMF, dayside reconnection contributes more to enhancements in cross polar cap potential compared to nightside reconnection. Recently, Boudouridis et al. (2021) studied the dynamic pressure effect on dayside and nightside reconnection using SuperDARN and compared it to MHD simulations. They observed an immediate response in the dayside reconnection rate, as well as a phased response in the nightside reconnection rate, which was delayed by ∼15–20 min.

Previous studies have explored the reconnection rate under various solar wind conditions, yet understanding its correlation with magnetopause motion has remained elusive due to limitations in simultaneous observations of both phenomena. Consequently, two primary questions persist: (1) how does the magnetopause position change in relation to changes in the reconnection rate? (2) what is the extent of the magnetopause movement that is attributed to reconnection itself, under the condition of steady solar wind dynamic pressure? These questions will be thoroughly addressed with the anticipated launches of the lunar environment heliospheric X-ray imager (Walsh et al. 2024) and solar wind–magnetosphere–ionosphere link explorer (SMILE; Branduardi-Raymont et al. 2018) in the near future. These missions aim to observe the magnetopause motion using soft X-rays to understand dayside reconnection modes. In anticipation of their launch, this paper employs empirical models of magnetopause location (Shue et al. 1998 and Lin et al. 2010) combined with SuperDARN observations, to investigate the relationship between magnetopause position and dayside reconnection rate. Section 2 describes the methodology in detail, including datasets and analysis procedures, and case studies are presented in Sect. 3. The ensuing discussion of our analysis results is provided in Sect. 4, followed by a comprehensive summary and conclusion in Sect. 5.

## Methodology

To compare the relationship between magnetopause position and reconnection rate, we defined the magnetopause position as the position of the subsolar magnetopause (hereafter ${\mathbf{R}}_{{\varvec{s}}}$). The reconnection rate (hereafter ${\mathbf{E}}_{{\varvec{r}}}$) was defined as the electric field measured at the OCB in the OCB-moving reference frame.

### Calculation of ${\mathbf{E}}_{{\varvec{r}}}$ by SuperDARN observation

SuperDARN measures ionospheric Doppler velocity and spectral width along multiple radial beams with a coarse latitudinal resolution (~ 0.3–0.4°). The common mode cadence for these measurements is set at 1–2 min (Greenwald et al. 1995; Chisham et al. 2007; Nishitani et al. 2019). For the present study, we utilized a SuperDARN radar located at Rankin Inlet (RKN; 72.6 $^\circ$ MLAT, – 26.4° MLON) in the northern hemisphere. The Altitude Adjusted Corrected Geomagnetic (AACGM) coordinate system (Shepherd 2014) was used to determine the locations of SuperDARN observations.

**Fig. 1:**
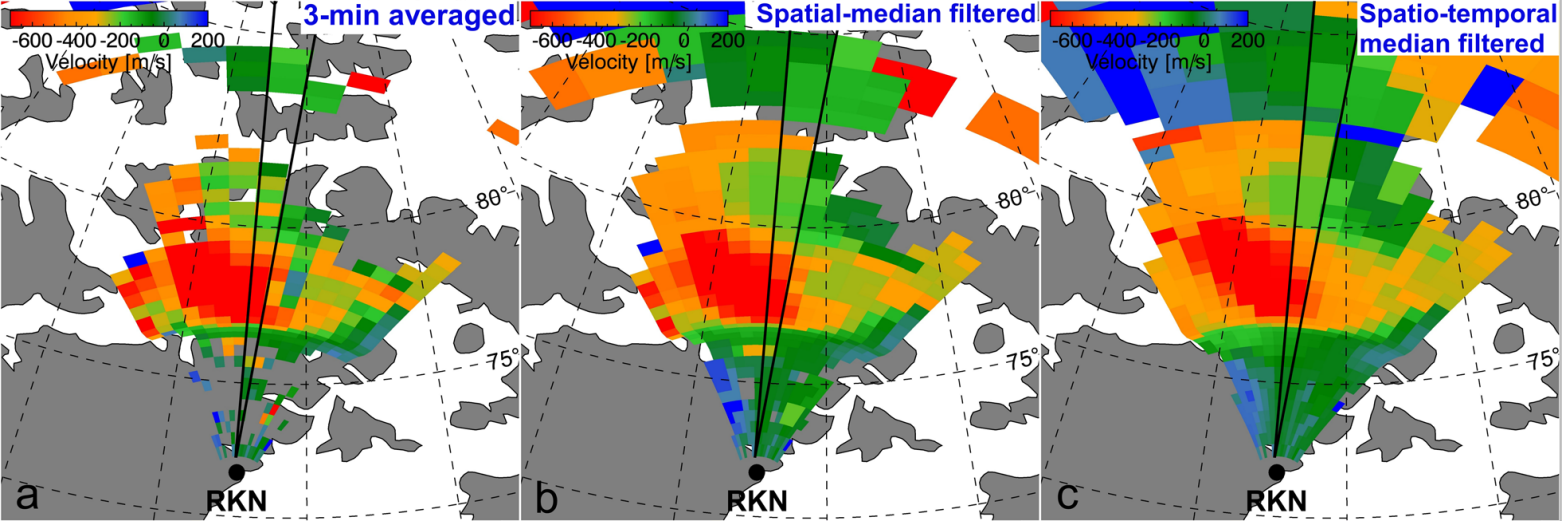
Doppler velocity measured by beams 0–15 of the RKN radar at 17:30 UT on 20 December 2017. **a** 3-min averaged raw data, **b** spatial-median filtered (**a**), and **c** both spatial- and temporal-median filtered (**a**). Black lines indicate the field of view of beam 7, and black dashed lines indicate the magnetic longitude and latitude calculated by AACGM

The reconnection on dayside and nightside is associated with the cross polar cap potential (or potential drop). By *Faraday's law*, the change in the open magnetic flux across the polar cap can be expressed as the integral around the OCB of the reconnection electric field in the reference frame of the OCB (e.g., Hubert et al. 2006; Comisso and Bhattacharjee 2016):

1 $$\vec{E} = \overrightarrow {{E_{i} }} + \overrightarrow {{V_{\rm o} }} \times \vec{B},$$

where $\overrightarrow{{E}_{i}}$ is the ionospheric electric field measured by an observer on the ground, $\overrightarrow{{V}_{\rm o}}$ is the velocity of the OCB obtained from SuperDARN measurements, and $\overrightarrow{B}$ denotes the magnetic field obtained from IGRF-13 under the assumption that magnetic field lines are frozen into plasma flowing perpendicular to the Earth’s magnetic field. $A$ localized reconnection rate (${E}_{\rm r}$) at a single point can be calculated in given $\overrightarrow{{V}_{\rm o}}$ and plasma flow velocity $\overrightarrow{{V}_{\rm p}}$ by assuming that $\overrightarrow{{V}_{\rm o}}$ is parallel or anti-parallel to $\overrightarrow{{V}_{\rm p}}$:

2 $$E_{\rm r} = \left( {V_{\rm o} - V_{\rm p} } \right)B,$$

where ${V}_{\rm p}$ can also be obtained from SuperDARN measurements. To deduce the temporal variation of ${E}_{\rm r}$, we averaged velocities over 3 min presuming that the reconnection varies smoothly during the 3 min. This method helps to mitigate spiking ${V}_{\rm o}$, which could arise due to temporal resolution or random errors. Subsequently, we employed a $3\times 3$ median filter for the central beam and two adjacent beams at each time step (spatial-median filter). This involved replacing each target echo with the median value of 9 points, including the target echo itself. We then applied another $3\times 3$ median filter across time and MLAT for each beam (temporal-median filter). This approach inspired by Chisham and Freeman (2003), aims to eliminate empty or isolated peak echoes. Figure 1 illustrates the data processing procedure. The geolocation of SuperDARN echoes was derived based on the virtual height model by Chisham et al. (2008b). Figure 1a shows a radar field-of-view plot presenting the 3-min averaged plasma velocity measured by the RKN radar at 17:30 UT on December 20, 2017 (Event 3 in this study). Figure 1b shows the flow velocity at the same time after applying the spatial-median filter across two adjacent beams. Finally, Fig. 1c exhibits the spatial- and temporal-median filtered flow velocities.

After averaging and filtering, we implemented a data binning of 0.5$^\circ$ MLAT. This allows us to capture the temporal variation of flow velocity and spectral width for the MLAT. Next, we identified the OCB at each time to obtain ${V}_{\rm o}$. The OCB has usually been defined as the equatorward boundary of 150–200 $\text{m}/\text{s}$ spectral width; regions poleward (equatorward) of it hosts open (closed) field lines (e.g., Chisham and Freeman 2003; Chisham et al. 2008a; Zou et al. 2019, 2022). In the present study, we defined the OCB as the equatorward boundary of $<span class='convertEndash'>120-130</span> \text{m}/\text{s}$ spectral width, where the largest latitudinal gradient of spectral width is located. The OCBs defined in this study match well with the boundary of strong poleward plasma flow velocities, as shown in Sect. 3 of this paper. Once the OCBs at each time are identified, the velocity of the moving OCB is

3 $${\mathbf{V}}_{{\varvec{o}}} = 110\user2{ }{\text{km}} \times \left( {{\varvec{OCB}}_{{{\varvec{t}} + 1}} - {\varvec{OCB}}_{{\varvec{t}}} } \right)/180\user2{ }\;{\text{s}}{.}$$

Here, 110 km represents the distance for 1° MLAT, ${{\varvec{O}}{\varvec{C}}{\varvec{B}}}_{{\varvec{t}}}$ and ${{\varvec{O}}{\varvec{C}}{\varvec{B}}}_{{\varvec{t}}+1}$ are the MLATs of OCB at time ${\varvec{t}}$ and ${\varvec{t}}+1$, respectively. ${\mathbf{V}}_{{\varvec{o}}}$ may consist of spikes due to the limited MLAT resolution (0.5°). Note that we also average ${\mathbf{V}}_{{\varvec{p}}}$ over $1^\circ$ latitude poleward of the OCB to minimize the possible uncertainties in SuperDARN measurements when determining the OCB (e.g., Chisham et al. 2005; Zou et al. 2022). Once ${\mathbf{V}}_{{\varvec{o}}}$ and ${\mathbf{V}}_{{\varvec{p}}}$ are determined, the temporal variation of ${\mathbf{E}}_{{\varvec{r}}}$ can be calculated using Eq. (2).

Since the plasma convection associated with reconnection depends on the IMF ${\text{B}}_{y}$, it is challenging to define OCB moving parallel to convection direction. In this study, we did not consider the angle between the plasma flow direction and the normal to the OCB in estimating ${\text{E}}_{\rm r}$ (e.g., Eq. (2) in Pinnock et al. 2003), which may lead to underestimation or overestimation of the reconnection flow speed normal to the OCB. Instead, we compare ${\mathbf{R}}_{{\varvec{s}}}$ with the average reconnection rate $\overline{{{\varvec{E}} }_{{\varvec{r}}}}$ estimated from the average $\overline{{{\varvec{V}} }_{{\varvec{o}}}}$ and $\overline{{{\varvec{V}} }_{{\varvec{p}}}}$ in regions where the line-of-sight of the beams aligns more closely with the convection flow and the OCB is clearly identifiable. For event 1, we averaged observations from beams 7, 9, and 10. For events 2 and 3, we averaged beams 4 to 7. For clarification on the direction of plasma convection relative to the SuperDARN line-of-sight, refer to Figure S1.

### Estimation of ${\mathbf{R}}_{{\varvec{s}}}$ by empirical models

#### Magnetopause models

The magnetopause models proposed by Shue et al. (1998) and Lin et al. (2010) are extensively used to determine the position of the magnetopause. Although empirical models may not precisely reproduce the absolute location of the magnetopause, they are the most readily available resources for capturing global magnetopause position.

The Shue et al. (1998) model (hereafter S98) estimates a 2-D magnetopause shape based on the empirical fit of magnetopause crossing data from several satellites. It uses IMF ${\text{B}}_{z}$ and solar wind dynamic pressure as input parameters and exhibits axisymmetric magnetopause with respect to the Sun–Earth line. It does not account for the magnetospheric cusp regions. Later, Lin et al. (2010) model (hereafter L10), building upon more extensive datasets of magnetopause crossings, offers a 3-D asymmetric magnetopause representation. Its coefficients are influenced by solar wind magnetic pressure and Earth’s magnetic dipole tilt angle, as well as solar wind dynamic pressure and IMF B_z_. L10 model can account for the hemispheric asymmetry of magnetopause and for the indentation around the cusp. Note that in this paper, the ${R}_{\rm s}$ defined by S98 and L10 models is a subsolar magnetopause on the Sun–Earth line. This can give us a rough idea about the magnetopause position relative to global magnetic reconnection rate.

#### Time-shift of ${\mathbf{R}}_{{\varvec{s}}}$

The solar wind propagation from the bow shock nose to the magnetopause and then to the ionosphere takes a few to ~ 20 min (e.g., Hairston and Heelis 1995; Etemadi et al. 1988; Connor et al. 2014; Bristow et al. 2022; Lockwood and McWilliams 2021). Given that empirical magnetopause models rely on OMNI solar wind data, which are propagated to the bow shock nose, adjusting the time shift of estimated ${R}_{\rm s}$ becomes essential for a comprehensive investigation of ${R}_{\rm s}$ in relation to the estimated $\overline{{{\varvec{E}} }_{{\varvec{r}}}}.$ To find the optimal propagation time-lag, we took the following steps assuming that the correspondence between ${R}_{\rm s}$ and $\overline{{{\varvec{E}} }_{{\varvec{r}}}}$ exists: (1) estimate ${R}_{\rm s}$ using the L10 model with NASA OMNI solar wind input from 30 h prior to initial enhancement of the SuperDARN plasma flow. (2) Calculate $\overline{{{\varvec{E}} }_{{\varvec{r}}}}$ using SuperDARN measurements for the reconnection period during which SuperDARN observes enhanced poleward plasma flows. (3) Calculate multiple correlation coefficients between $\overline{{{\varvec{E}} }_{{\varvec{r}}}}$ and ${R}_{\rm s}$ by shifting ${R}_{\rm s}$ by 3-min steps. (4) Determine the optimal time-lag between $\overline{{{\varvec{E}} }_{{\varvec{r}}}}$ and ${R}_{\rm s}$ by selecting a time shift that gives the maximum of absolute correlation coefficient. Finally, shift the ${R}_{\rm s}$ for the time step calculated from (4) and then compare it with $\overline{{{\varvec{E}} }_{{\varvec{r}}}}$. Note that, the optimum time-lag changes by $\pm 3$ min if we use the S98 model to calculate ${R}_{\rm s}$. However, there is no substantial variance in the correlation coefficient within $\pm 3$-min time-lag when opting for the S98 model.

## Case studies

**Fig. 2:**
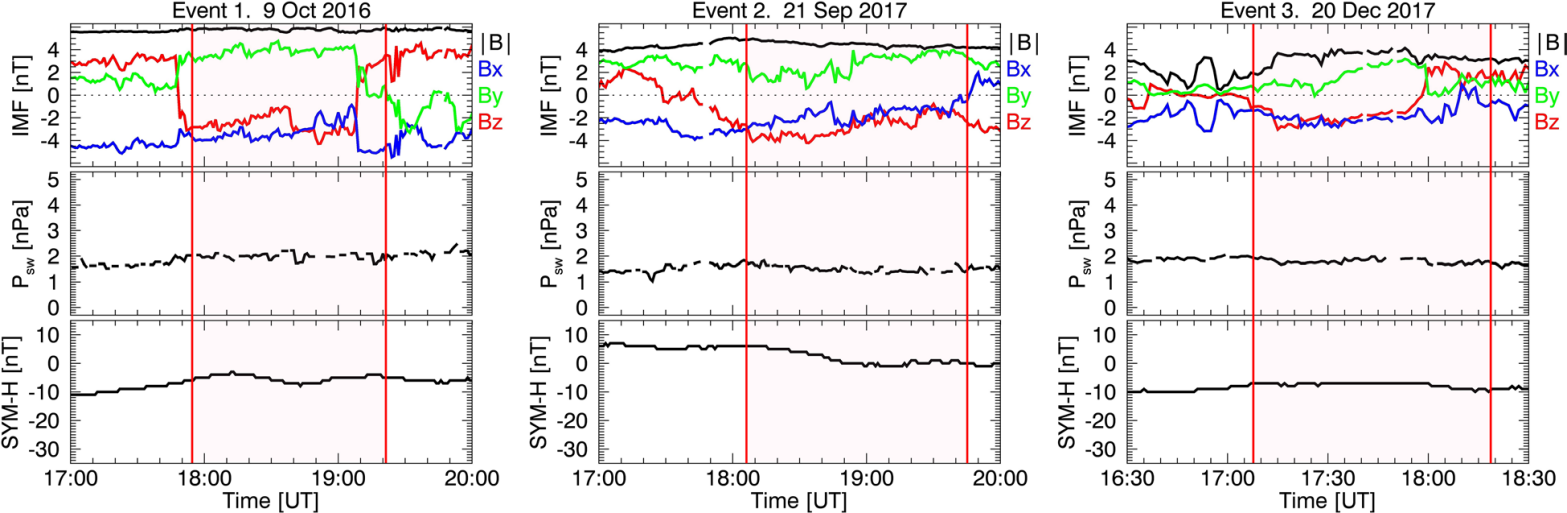
NASA OMNI IMF, solar wind dynamic pressure (${\mathbf{P}}_{\mathbf{s}\mathbf{w}}$), and SYM-H index for three events. Red vertical lines indicate the event period

Under the assumption that the enhancement of the ionospheric poleward flow during southward IMF is primarily caused by dayside reconnection, we identified three events from SuperDARN measurements that occurred on 9 October 2016, 21 September 2017, and 20 December 2017. Figure 2 shows the OMNI IMF, solar wind dynamic pressure (${P}_{\text{sw}}$), and SYM-H index for each event (1-min time resolution). The red vertical lines indicate the event periods analyzed to study the relationship between ${R}_{\rm s}$ and ${E}_{\rm r}$ (SuperDARN event periods). These events occurred during southward IMF and quasi-steady ${P}_{\text{sw}}$ (< 2 nPa) fluctuating within 1 nPa. SYM-H exceeding – 13 nT represents a geomagnetically quiet period, with the ring current contribution to ${R}_{\rm s}$ change being minimal. Note that the time-lag between the bow shock nose and ionosphere will be considered to estimate ${R}_{\rm s}$. Detailed observations and analysis of the results are presented in the subsequent subsections.

### Event 1: 9 October 2016

**Fig. 3:**
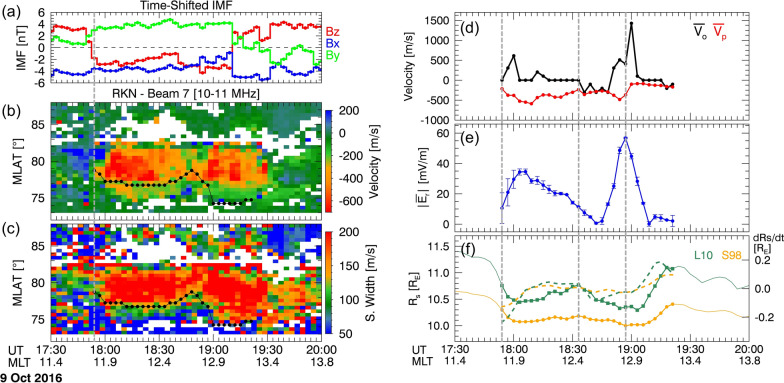
Plots depicting the event of October 9, 2015. **a** Time-shifted OMNI IMF. **b, c** Range plots of Doppler velocity and spectral width measured by RKN radar beam 7. The black dotted lines indicate OCB. **d** Average velocities of OCB ($\overline{{{\varvec{V}} }_{{\varvec{o}}}}$) and plasma flow ($\overline{{{\varvec{V}} }_{{\varvec{p}}}}$). **e** Average reconnection rate ($\left|\overline{{{\varvec{E}} }_{{\varvec{r}}}}\right|$) for the OCB moving reference frame. **f** Magnetopause position (${\mathbf{R}}_{{\varvec{s}}}$) estimated by Shue et al. (1998) and Lin et al. (2010) magnetopause

The first event occurred after an abrupt IMF turning from northward to southward. Figure 3a shows the IMF with a 3-min cadence. In this event, a 3-min time-shift is applied based on the method described in Sect. 2.2.2. IMF ${\text{B}}_{z}$ remained consistently northward until 17:51 UT, after which it turned southward and persisted until 19:09 UT. During this period, ${B}_{x}$ exhibited negative polarity while ${B}_{y}$ remained steadily positive. Subsequently, at 19:12 UT, ${B}_{z}$ rapidly turned northward, coinciding with decreases in ${\text{B}}_{x}$ and ${\text{B}}_{y}$.

Figure 3b, c shows plasma flow velocity and spectral width measured by beam 7 (central beam, black lines in Fig. 1) of the RKN radar, respectively. The collected data include echoes from signals transmitted within the 10–11 MHz frequency range. The strong poleward plasma convection (red; negative values) in Fig. 3b begins at 17:54 UT, following the north-to-southward turning of ${\text{B}}_{z}$, and persists until 19:30 UT. Note that SuperDARN data is averaged over 3-min intervals, thus the timestamp 17:54 UT incorporates data between 17:54 and 17:56 UT. The convection speed slightly decreased around 18:30 UT, coinciding with the weakening of the southward IMF, and the subsequent northward turning of ${B}_{z}$. The increase in spectral width, as shown in Fig. 3c, aligns with the period of rapid plasma convection. Therefore, we define the time interval between 17:54 UT and 19:21 UT as the reconnection period during which we compare $\overline{{{\varvec{E}} }_{{\varvec{r}}}}$ with ${R}_{\rm s}$.

OCB is estimated using the method in Sect. 2.1 and is displayed with black dotted lines in Fig. 3b, c. Initially located at 79° MLAT, the OCB shifted between 73.5° and 79° MLAT during the reconnection period, while beam 7 of the RKN radar was positioned around noon. The strong poleward flows were observed between 19:21 and 19:30 UT, but we do not analyze this period as we cannot define the location of the OCB from the spectral width. Note that the time discrepancy between the northward turning of IMF (19:12 UT) and the weakening of flow velocity (19:30 UT) suggests that the propagation time from the bow shock nose to the ionosphere was inconsistent during our period of interest.

Figure 3d shows the average $\overline{{{\varvec{V}} }_{{\varvec{o}}}}$ (black dotted line) and $\overline{{{\varvec{V}} }_{{\varvec{p}}}}$ (red dotted line) of beams 7, 9, and 10 during reconnection. A positive $\overline{{{\varvec{V}} }_{{\varvec{o}}}}$ indicates the OCB moving equatorward, while a negative $\overline{{{\varvec{V}} }_{{\varvec{o}}}}$ signifies movement towards the pole. Throughout the reconnection period, the OCB predominantly exhibited equatorward motion, with a brief poleward motion between 18:33 and 18:48 UT. This poleward motion may be attributed to the weakening of the southward IMF around 18:30 UT. The $\overline{{{\varvec{V}} }_{{\varvec{p}}}}$ was consistently negative, indicating reconnection-driven poleward convection flows. Figure 3e shows $\overline{{{\varvec{E}} }_{{\varvec{r}}}}$ in the OCB moving reference frame, with error bars representing the standard deviation. To mitigate the rapidly changing $\overline{{{\varvec{E}} }_{{\varvec{r}}}}$, we applied a second-order Savitzky–Golay smoothing filter (Savitzky and Golay 1964) to $\overline{{{\varvec{E}} }_{{\varvec{r}}}}$.

Now, we examine the position of the magnetopause in relation to the reconnection rate. Figure 3f shows ${R}_{\rm s}$ estimated by the L10 (green line with squares) and the S98 (orange line with circles) models, respectively. The dashed lines represent the $\text{d}{R}_{s}/\text{d}t$. Before 17:54 UT, the ${R}_{\rm s}$ of the L10 (S98) were ~ 11.3 ${R}_{\text{E}}$ (~ 10.5 ${R}_{\text{E}}$) and decreased to ~ 10.5 ${R}_{\text{E}}$ (~ 10 ${R}_{\text{E}}$) by 18:00 UT, following the southward turning of the IMF. Meanwhile, $\overline{{{\varvec{E}} }_{{\varvec{r}}}}$ increased to 30 mV/m. Subsequently, ${R}_{\rm s}$ shifted sunward while $\overline{{{\varvec{E}} }_{{\varvec{r}}}}$ gradually decreased until 18:33 UT (the second vertical dashed line). Following this, ${R}_{\rm s}$ moved earthward again, while $\overline{{{\varvec{E}} }_{{\varvec{r}}}}$ showed an overall increase until 18:57 UT (the third vertical dashed line). Eventually, ${R}_{\rm s}$ returned to its initial position, with $\overline{{{\varvec{E}} }_{{\varvec{r}}}}$ dropping to 0. In summary, the variation in $\overline{{{\varvec{E}} }_{{\varvec{r}}}}$ exhibits an anti-correlation with ${R}_{\rm s}$. The $\text{d}{R}_{s}/\text{d}t$ fluctuated between $\pm 0.2$ and partially showed an anti-correlation with $\overline{{{\varvec{E}} }_{{\varvec{r}}}}$ (18:06–18:33 UT, 19:00–19:21 UT).

### Event 2: 21 September 2017

**Fig. 4:**
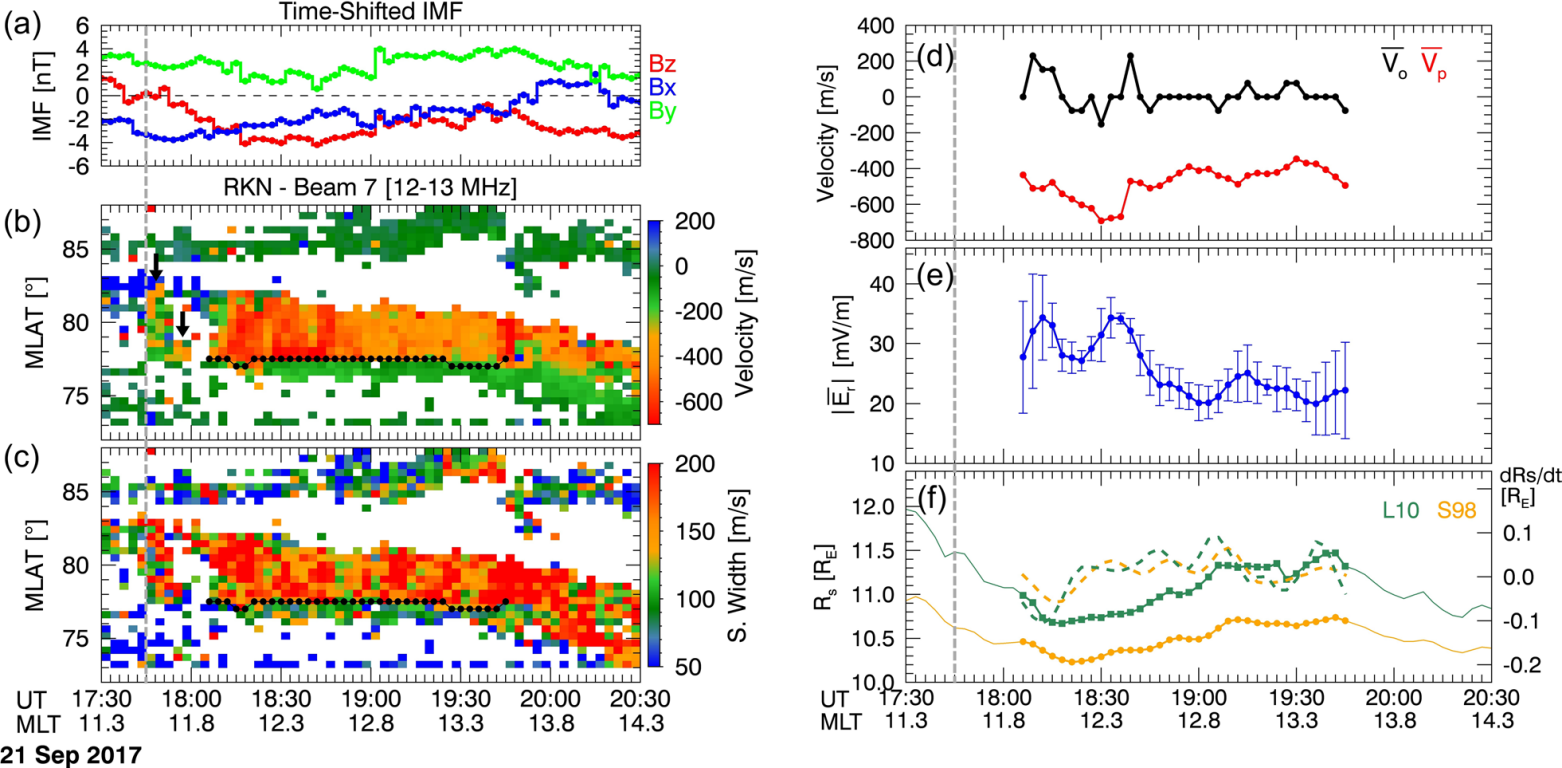
Plots depicting the event of September 21, 2017, in the same format as Figure 3

Event 2 also occurred after a north-to-southward turning of the IMF, but its transition occurred more slowly compared to event 1. Figure 4a shows the IMF shifted by 9 min to account for the propagation of solar wind/IMF impact from the bow shock nose to the ionosphere. IMF ${\text{B}}_{z}$ was northward for ~ 30 min before 17:36 UT and fluctuated from north to southward until 18:00 UT. Between 17:45 UT and 18:00 UT, poleward flow enhancements of $> -300 \text{m}/\text{s}$ were observed at 77.5°–82° MLAT (black arrows), as shown in Fig. 4b. These sporadic flow enhancements are likely due to reconnection while the IMF ${\text{B}}_{z}$ was temporarily southward at 17:39 UT. The time discrepancy between the southward IMF and flow enhancement may be due to inconsistent propagation time, similar to Event 1. After 18:00 UT, IMF ${\text{B}}_{z}$ continued to stay southward and the SuperDARN RKN radar continuously observed strong poleward ionospheric plasma flow. The SuperDARN OCB was almost fixed and shifted slightly by 0.5° from 77° MLAT around 18:20 UT and 19:30 UT while it crossed noon, as shown in Fig. 4b, c. Note that, in this event, we analyze data between 18:06 UT and 19:45 UT to avoid potential errors in the location of the OCB caused by sparse radar signals and uncertainties beyond this period.

Now, we compare $\overline{{{\varvec{E}} }_{{\varvec{r}}}}$ and ${R}_{\rm s}$ from Fig. 4d–f. During this event, $\overline{{{\varvec{V}} }_{{\varvec{o}}}}$ changed between $-150$ and $+250$ km/s, indicating that the reconnection rate primarily depends on $\overline{{{\varvec{V}} }_{{\varvec{p}}}}$ increasing up to $-700$ km/s. ${R}_{\rm s}$ for both models moved back and forth before 18:00 UT, likely due to reversals of IMF ${B}_{z}$. Subsequently, ${R}_{\rm s}$ moved earthward until 18:15 UT for L10 and 18:21 UT for S98 with an increase in southward IMF. Similar to event 1, ${R}_{\rm s}$ moved earthward when $\overline{{{\varvec{E}} }_{{\varvec{r}}}}$ increased and shifted sunward when $\overline{{{\varvec{E}} }_{{\varvec{r}}}}$ decreased. Occasionally, $\overline{{{\varvec{E}} }_{{\varvec{r}}}}$ and ${R}_{\rm s}$ show different behaviors such as increases in $\overline{{{\varvec{E}} }_{{\varvec{r}}}}$ with increasing ${R}_{\rm s}$ around 18:30 UT. However, the overall trend of $\overline{{{\varvec{E}} }_{{\varvec{r}}}}$ showed an anti-correlation with the change in ${R}_{\rm s}$. The $\text{d}{R}_{s}/\text{d}t$ fluctuated between $\pm 0.1$ and generally showed an anti-correlation with $\overline{{{\varvec{E}} }_{{\varvec{r}}}}$.

### Event 3: 20 December 2017

**Fig. 5:**
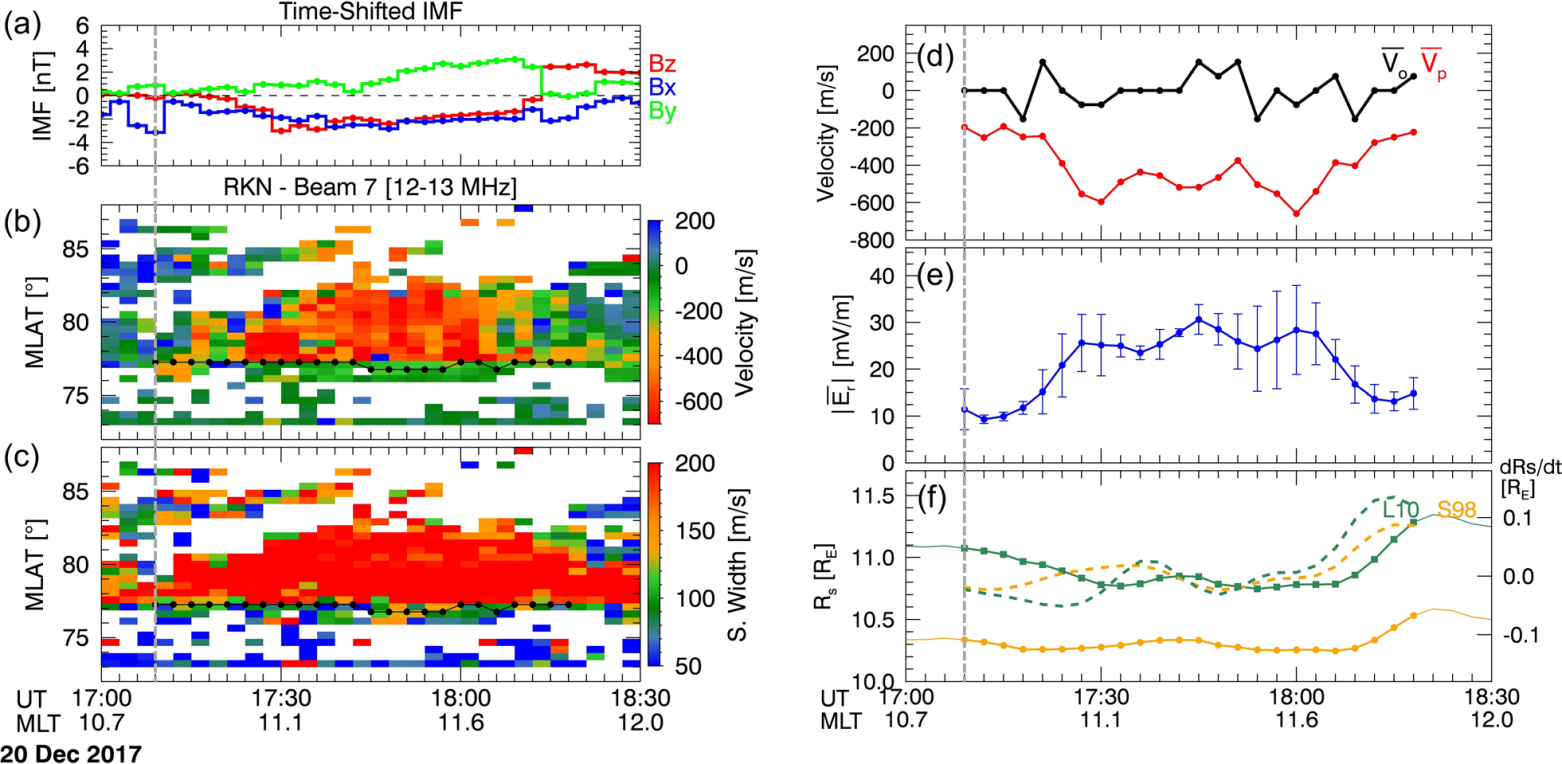
Plots depicting the event of December 20, 2017, in the same format as Figure 3

Event 3 occurred following a gradual change of IMF *B*_z_ from 0 to – 2 nT. Figure 5a shows 15-min time-shifted IMF. Until 17:09 UT (grey vertical line), IMF ${\text{B}}_{x}$ and ${\text{B}}_{y}$ were dominant, with IMF ${\text{B}}_{z}$ exhibiting 0 or weakly southward. Figure 5b shows weak enhancements in ionospheric flows ($-180\text{ to}-270\text{ m}/\text{s}$) during 17:09–17:24 UT, possibly related to the weak southward IMF. Subsequently, strong poleward plasma flows ($>-890\text{ m}/\text{s}$) were observed until 18:18 UT during a period of strong and continuous southward IMF. The SuperDARN OCB remained almost fixed at 77° MLAT and shifted slightly by 0.5° from 77° MLAT between 17:45 UT and 18:06 UT. Similar to Event 2, $\overline{{{\varvec{V}} }_{{\varvec{o}}}}$ exhibited weaker changes compared to $\overline{{{\varvec{V}} }_{{\varvec{p}}}}$, indicating that the reconnection rate primarily depends on $\overline{{{\varvec{V}} }_{{\varvec{p}}}}$.

In Fig. 5e, f, similar to previous events, both ${R}_{\rm s}$ and $\text{d}{R}_{s}/\text{d}t$ overall showed anti-correlation with $\overline{{{\varvec{E}} }_{{\varvec{r}}}}$. ${R}_{\rm s}$ of L10 exhibited an earthward moving by 0.3–0.4 ${\text{R}}_{\text{E}}$ during the reconnection, while the S98 model showed subtle changes. They moved back and forth during the reconnection period and finally returned to their initial locations at 18:12 UT after the northward turning of the IMF.

## Discussion

### Relation between ${\mathbf{R}}_{{\varvec{s}}}$ and ${\mathbf{E}}_{{\varvec{r}}}$

**Fig. 6:**
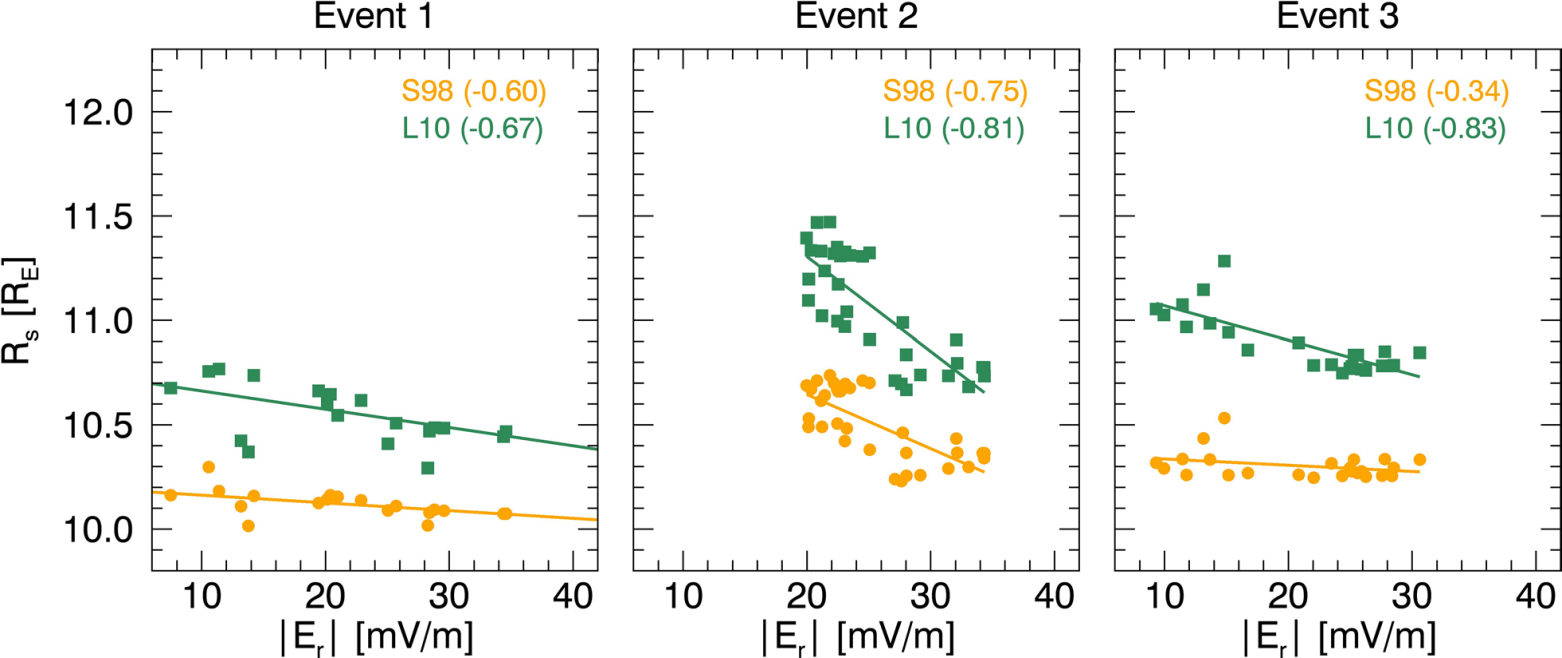
Scatterplots of ${\mathbf{R}}_{{\varvec{s}}}$ vs $\left|\overline{{{\varvec{E}} }_{{\varvec{r}}}}\right|$ of three events, with correlation coefficients indicated in brackets

All events in this study exhibited a reasonably strong anti-correlation between ${R}_{\rm s}$ and $\overline{{{\varvec{E}} }_{{\varvec{r}}}}$. Figure 6 presents scatter plots with correlation coefficients (*r*) for each event. ${R}_{\rm s}$ and $\overline{{{\varvec{E}} }_{{\varvec{r}}}}$ show an anti-correlation with $\left|r\right|>0.60$ except for Event 3 when S98 is utilized.

Note that it is challenging to quantify the magnetopause motion, i.e., $\text{d}{R}_{\text{s}}/\text{d}t$, in relation to varying reconnection rates. This difficulty arises because the empirical magnetopause model may not capture the sequential changes in magnetopause position over a specific period, resulting in fluctuating or noisy $\text{d}{R}_{\text{s}}/\text{d}t$. Derivative of a noisy signal is much noisier, which can possibly overwhelm any valid trend (e.g., Yip et al. 2020, Fig. 1). Despite these challenges, $\text{d}{R}_{\text{s}}/\text{d}t$ overall showed a moderate anti-correlation with $\overline{{\mathbf{E} }_{\mathbf{r}}}$, although it exhibited a partially positive correlation in Event 1.

**Fig. 7:**
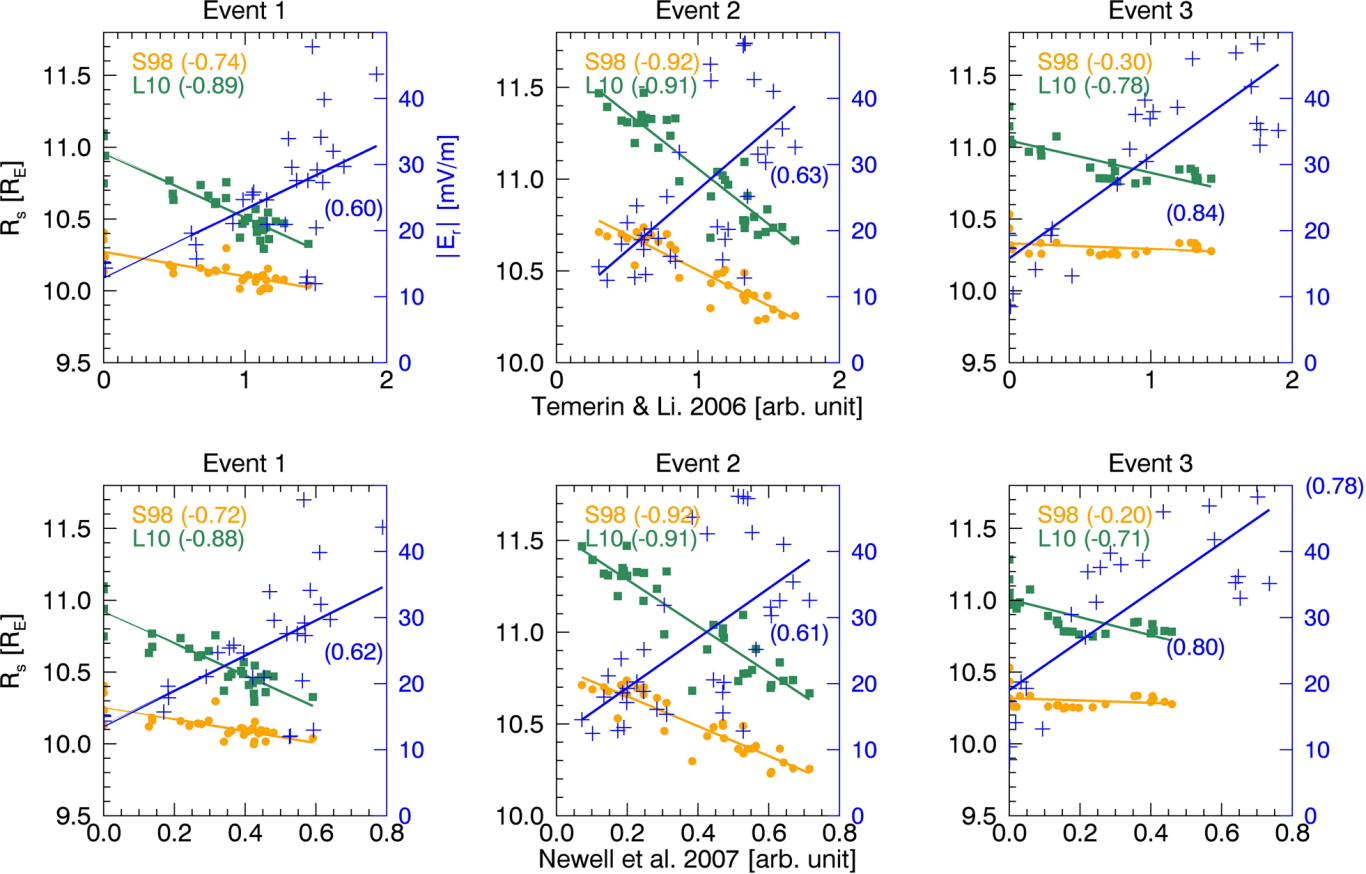
Scatterplots of ${R}_{\rm s}$ and $\left|\overline{{{\varvec{E}} }_{{\varvec{r}}}}\right|$ vs coupling efficiencies based on Temerin and Li (2006) and Newell et al. (2007), with correlation coefficients indicated in brackets

The empirical coupling functions in previous references (e.g., Perrault and Akasofu 1978; Kan and Lee 1979; Vasyliunas et al. 1982; Temerin and Li 2006; Newell et al. 2007) represent the efficiency of interaction between the solar wind and the magnetosphere. While these efficiencies do not precisely represent reconnection efficiency, we consider them as a proxy for dayside reconnection. We calculate the correlation coefficients between this efficiency, derived from the coupling functions, and the ${R}_{\rm s}$ and ${E}_{\rm r}$. We select two empirical coupling functions: one by Temerin and Li (2006, ${n}^{1/2}{v}^{2}{B}_{T}{\text{sin}}^{6}({\theta }_{c}/2)$) and the other by Newell et al. (2007, ${v}^{4/3}{{B}_{T}}^{2/3}{\text{sin}}^{8/3}({\theta }_{c}/2)$). Both models utilize solar wind input parameters such as solar wind density (*n*), velocity (*v*), IMF (${B}_{T}$), and IMF clock angle (${\theta }_{c}$). Figure 7 presents scatter plots of unitless coupling efficiencies versus ${R}_{\rm s}$ and versus $\overline{{{\varvec{E}} }_{{\varvec{r}}}}$, along with their respective correlation coefficients. ${R}_{\rm s}$ exhibited reasonable anti-correlation with the empirical coupling efficiencies ($\left|r\right|$> 0.71), except for the S98 model ($\left|r\right|$< 0.3) in event 3. $\overline{{{\varvec{E}} }_{{\varvec{r}}}}$ also showed reasonable correlation with coupling efficiencies ($\left|r\right|$> 0.6). The uncertainties in estimating the propagation time between the bow shock nose and ionosphere, or in OCB identification, as discussed in Sect. 4.3, can result in less direct correspondence between reconnection rate and coupling efficiency.

### Differences in empirical models

The S98 model estimated ${R}_{\rm s}$ to be closer to Earth compared to the L10 model. In contrast to a study by Case and Wild (2013), which compared magnetopause positions between Cluster observations and model estimations and found that the S98 model tends to overestimate ${R}_{\rm s}$ while the L10 model tends to underestimate it, our study presents inconsistent results. However, in this paper, our focus is on the qualitative trend of ${R}_{\rm s}$ changes, and we refrain from attempting a quantitative evaluation of the models, although ${R}_{\rm s}$ of S98 model showed lower correlation compared to L10 model.

### Uncertainty in OCB identification

Interpreting events 2 and 3 poses a challenge as both ${R}_{\rm s}$ and ${\mathbf{E}}_{{\varvec{r}}}$ change, while the OCB extracted from a specific beam of SuperDARN data remained overall fixed, as depicted in Figs. 4b, c and 5b, c. Normally, changes in ${\mathbf{E}}_{{\varvec{r}}}$ would suggest shifts in the magnetopause and its ionospheric footprint (OCB). However, this expectation contradicts the observations in Figs. 4 and 5. We attribute this inconsistency to the potential absence of a direct correlation between OCB and ${R}_{\text{s}}$ motion in events 2 and 3. It appears that the reconnection rate may only marginally depend on the OCB location for events 2 and 3, whereas event 1 indicates some level of synchronized variation between ${V}_{\text{o}}$ and ${\text{R}}_{\text{s}}$. Further investigation is necessary to verify whether alterations in the magnetopause location are accurately detected in the ionosphere.

The RKN SuperDARN radar is typically located deep within the polar cap, introducing uncertainty in observing echoes transmitted from the F-region to the E-region. There can be a distinct difference in plasma irregularities between E- and F-regions, causing the boundary to be an artifact of the system; two regions of echoes from different altitudes that appear adjacent to each other along the beam (e.g., Chisham and Pinnock 2002), resulting in uncertainty in OCB identification. In addition, the inherent limitations of the thresholding method utilized to define the OCB (i.e., $130 \text{m}/\text{s}$ in spectral width), with a latitudinal resolution of 0.5°, may be associated with the discrepancy between OCB and ${R}_{\rm s}$ motion. However, there is no universal agreement on the SuperDARN spectral width threshold for OCB identification. If optimal methods are employed to define OCBs consistently over time, they might exhibit temporal variations more closely aligned with the model ${R}_{\rm s}$. Previous studies adopted diverse methods to identify the OCB. For instance, particle precipitation signatures observed by low-Earth orbit satellites (Newell et al. 2007; Newell and Meng 1989), auroral images observed by all-sky imager (Blanchard et al. 1995, 1997; Sandholt et al. 1998), electron density signatures measured by incoherent scatter radar (Beaujardiѐre et al. 1994; Blanchard et al. 1996), field-aligned current undulation (Xiong and Lühr 2014), and Doppler spectral width measured by SuperDARN (Baker et al. 1995; Chisham and Freeman 2003) are used to identify the OCB. In the future, comparing coordinated observations of the magnetopause and OCB, employing variable thresholds for OCB identification, with multiple instruments such as auroral images, energetic particle precipitation, and SuperDARN, could help identify a better (or more universally applicable) threshold for SuperDARN observation. We also attempted to reduce the latitudinal resolution to 0.3°. While OCBs slightly changed at this resolution in both Event 2 and Event 3 (not shown here), the difference is within the error bar and did not alter our overall result.

### Azimuthal extent of reconnection

Our comparison involved assessing the ${R}_{\rm s}$ against SuperDARN ${\mathbf{E}}_{{\varvec{r}}}$ estimated at a time varying MLT, i.e., RKN SuperDARN OCB location may experience temporal shifts. In addition, all events in this study occurred during positive IMF ${B}_{y}$; thus, the line-of-sight of SuperDARN may not be located in the anti-sunward flow region if reconnection is localized. Despite the possibility of temporal misalignment in MLT, our chosen approach remains reasonable because the reconnection at the magnetopause can span wide regions from dawn to dusk (e.g., Chisham et al. 2004; Phan et al. 2006; Shepherd and Ruohoniemi 2000), and the anti-sunward flow can extend across a broad MLT range.

**Fig. 8:**
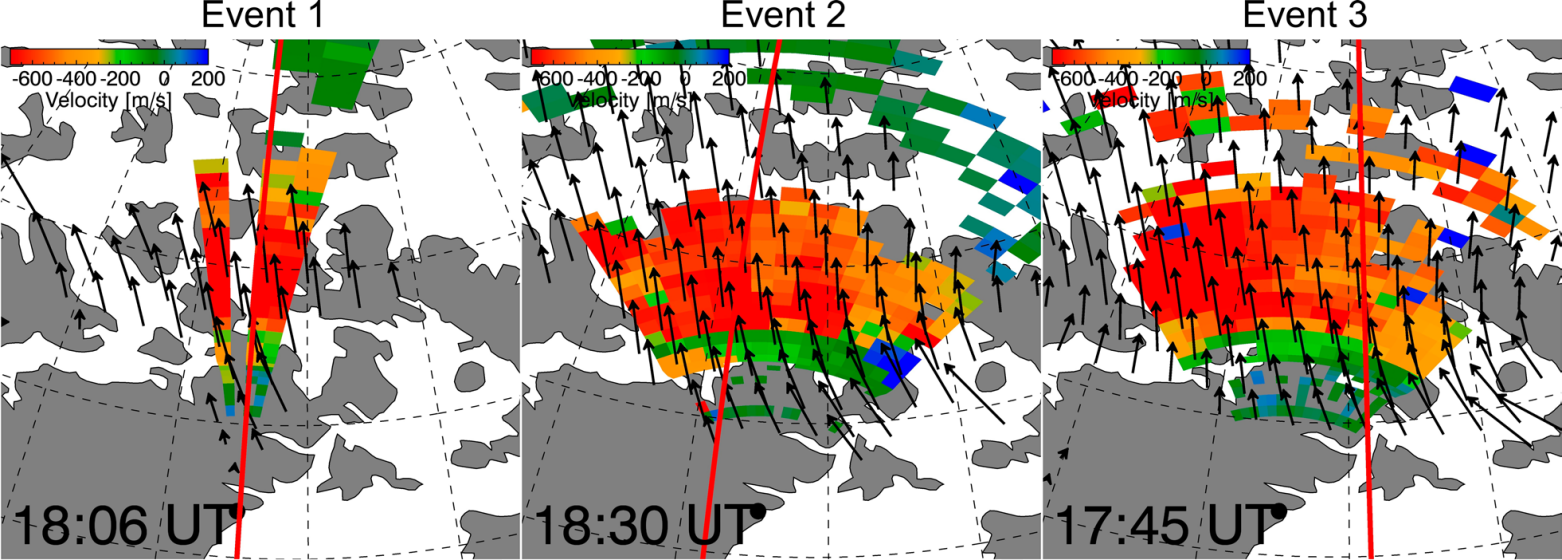
Line-of-sight plasma flow velocity superimposed with flow vector for three events at specific times. The red vertical line indicates the location of local noon

Figure 8 shows line-of-sight plasma flow velocity superimposed with flow vector for three events at specific times. The flow velocity vectors were obtained by merging line-of-sight measurements within the overlapping field of view of the radars (Ruohoniemi and Baker 1998). The red vertical line indicates the location of local noon, and the complete period plot during the event is presented in Figure S1. Throughout all event periods, the anti-sunward flows are predominantly observed over the line-of-sight of the beams that we used for averaging, indicating that the RKN radar observed ionospheric plasma flows related to reconnection. Furthermore, SuperDARN observed poleward flow enhancements in the prenoon-to-postnoon sector following north-to-southward IMF turnings. This observation suggests that the convection enhancements are likely caused by dayside reconnection near local noon. Therefore, the slight MLT mismatch between ${R}_{\rm s}$ and ${\mathbf{E}}_{{\varvec{r}}}$ does not compromise our conclusion regarding the relationship between ${R}_{\rm s}$ and ${\mathbf{E}}_{{\varvec{r}}}$.

### Effect of nightside reconnection

**Fig. 9:**
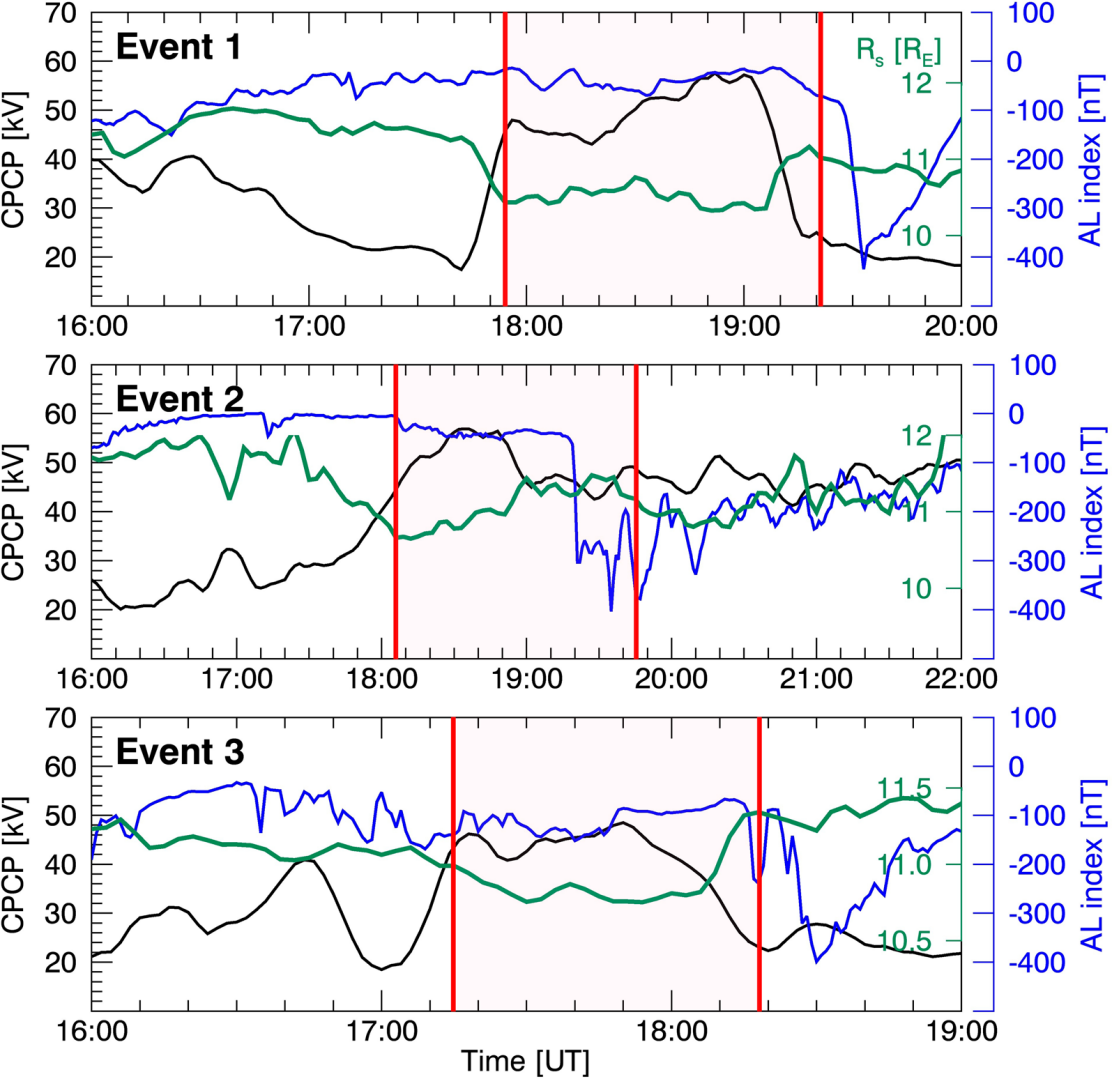
Cross polar cap potential (black), AL index (blue), and ${\mathbf{R}}_{{\varvec{s}}}$ estimated by the L10 model (green). Red vertical lines indicate the event period

Since the IMF were temporarily southward preceding our events (~ 80 min for event 1, 60 min for event 2, and 25 min for event 3), dayside ${R}_{\rm s}$ is influenced not only by dayside reconnection but also by nightside reconnection (e.g., Boudouridis et al. 2011, 2021). To investigate whether nightside reconnection contaminates the relationship between ${R}_{\rm s}$ and the dayside reconnection rate (${\mathbf{E}}_{{\varvec{r}}}$), Fig. 9 presents the cross-polar cap potential (CPCP, black lines) and the OMNI AL index superimposed with ${R}_{\rm s}$ from L10 model (magenta lines). The pink shading represents event periods. The CPCP has been used as a proxy for ionospheric convection strength associated with both dayside and nightside reconnection (e.g., Milan et al. 2003; Hubert et al. 2006), while the AL index represents nightside geomagnetic activity associated with substorms, i.e., nightside reconnection (e.g., Gjerloev et al. 2004; Partamies et al. 2013; Pulkkinen et al. 2014). In Events 1 and 3, the CPCP increased above 40 kV as ${R}_{\rm s}$ begins to decrease at ~ 17:50 UT and ~ 17:10 UT, respectively. Conversely, it decreased as ${R}_{\rm s}$ starts to increase at ~ 19:10 UT and ~ 18:05 UT, respectively, reaching ~ 20 kV. The AL index remained nearly constant during these events, implying that ${R}_{\rm s}$ is primarily influenced by dayside reconnection. Event 2 exhibited CPCP increases when ${R}_{\rm s}$ decreases. However, the AL index abruptly dropped after 19:20 UT, indicating that nightside reconnection may thereafter impact the ${R}_{\rm s}$ location and potentially contaminate the anti-correlation relationship between ${R}_{\rm s}$ and ${\mathbf{E}}_{{\varvec{r}}}$. Nevertheless, during other periods of constant AL, we consistently observed anti-correlation relationships between ${R}_{\rm s}$ and $\overline{{{\varvec{E}} }_{{\varvec{r}}}}$.

## Summary and conclusion

In this study, we compared the model magnetopause position with the SuperDARN reconnection rate during periods of quasi-steady solar wind pressure to investigate changes in the magnetopause position in relation to reconnection rates. Our approach involved estimating ${R}_{\rm s}$ from empirical models, incorporating time shifts to account for the propagation of solar wind/IMF impacts from the bow shock nose to the ionosphere. We then compared the model ${R}_{\rm s}$ with $\overline{{{\varvec{E}} }_{{\varvec{r}}}}$ derived from SuperDARN observations. All the events showed that $\overline{{{\varvec{E}} }_{{\varvec{r}}}}$ generally increased as the magnetopause located closer to Earth and vice versa. In addition, our result confirmed that $\overline{{{\varvec{E}} }_{{\varvec{r}}}}$ increased with an increase in the empirical coupling efficiency between solar wind and magnetosphere.

At present, direct comparisons between in-situ observations at the magnetopause and ionospheric responses face challenges due to the frequent spatio-temporal variation of the magnetopause, despite multiple spacecraft crossings. Looking ahead, with the forthcoming launch of missions such as LEXI and SMILE, we anticipate more accurate and seamless magnetopause positions to be extracted from their soft X-ray images of the Earth’s boundary layer. This will provide an opportunity to observationally test the methodology suggested in this study, allowing for a deeper understanding of magnetopause dynamics and their relationship with reconnection and ionospheric responses.

## Supplementary Information

Additional File 1: Figure S1. Line-of-sight plasma velocity within the radar field of view at the time intervals:17:54–19:21 UT during Event 1,18:06–19:45 UT during Event 2, and17:15–18:18 UT during Event 3. Arrows indicate velocity vectors inferred from multiple radars.

## Data Availability

The SuperDARN data in FITACF format, which includes spectral width and Doppler plasma velocity, was obtained online from (http://superdarn.ca). The geolocation of SuperDARN echoes can be obtained using the Space Physics Environment Data Analysis System (SPEDAS, Angelopoulos et al. 2019) version 6.0. The relevant directory, located at \projects\erg\ground\radar\superdarn\sdfovlib, contains the data as IDL save files (https://spedas.org/blog/). The convection flow data, provided by Virginia Tech, can be accessed at ‘https://zenodo.org/records/10940083’. The Solar wind, SYM-H, and AL indices were downloaded from the NASA OMNI web database (https://omniweb.gsfc.nasa.gov).

## Abbreviations

AACGM: Altitude-adjusted corrected geomagnetic coordinates
IMF: Interplanetary magnetic field
OCB: Open–closed magnetic field line boundary
SuperDARN: Super dual auroral radar network
RKN: Rankin inlet
LEXI: Lunar environment heliospheric X-ray imager
SMILE: Solar wind–magnetosphere–ionosphere link explorer
MLAT: Magnetic latitude
MLT: Magnetic local time
MHD: Magnetohydrodynamic
