# Supplementary figures and images for: Relation between magnetopause position and reconnection rate under quasi-steady solar wind dynamic pressure
Source: Earth Planets Space. 2024 Dec 18;76(1):165. doi: 10.1186/s40623-024-02101-9 (PMC11655602; doi:10.1186/s40623-024-02101-9)

Figure S1

(a) Event 1 (17:54-19:21 UT)

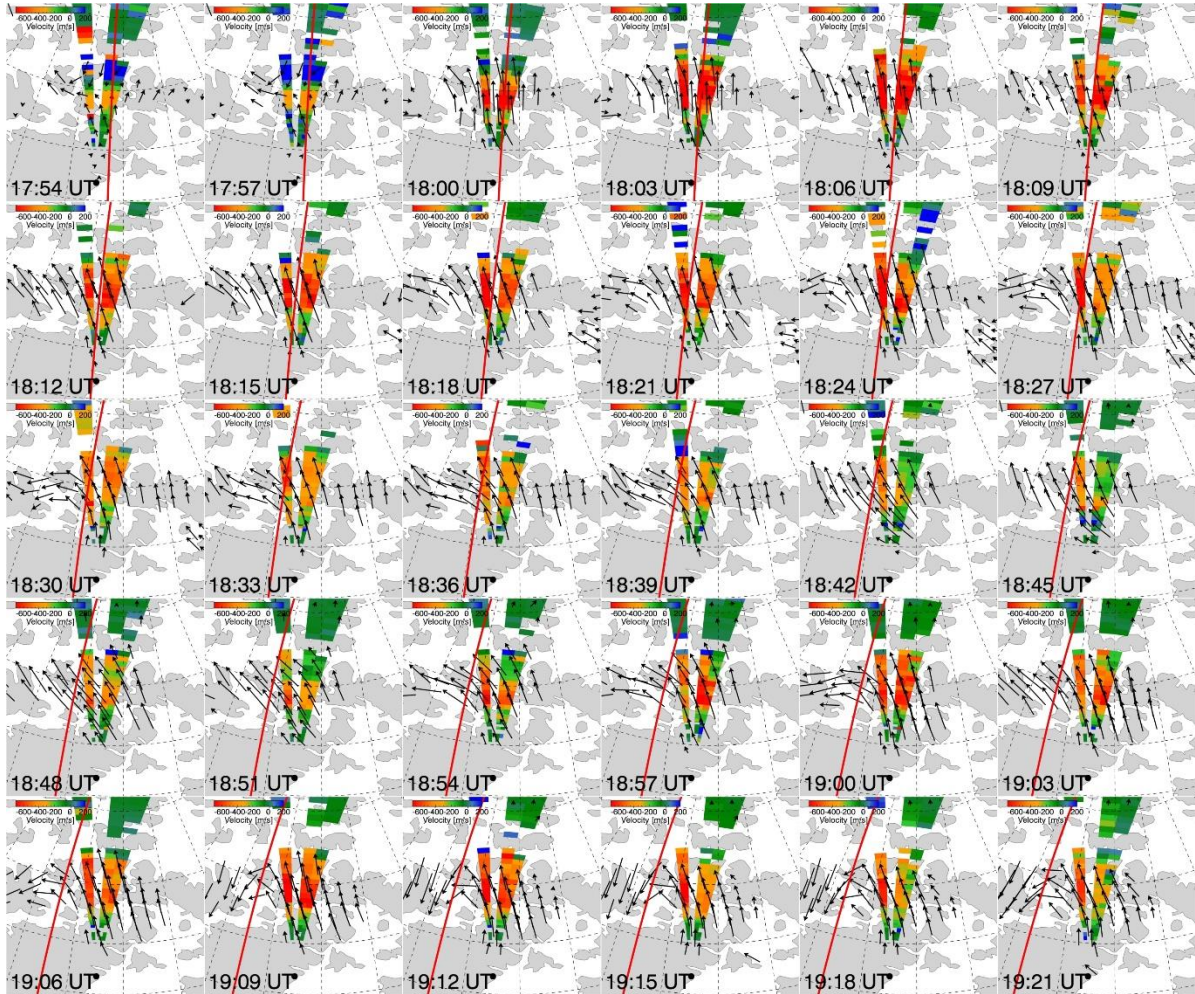

(b) Event 2 (18:06-19:45 UT)

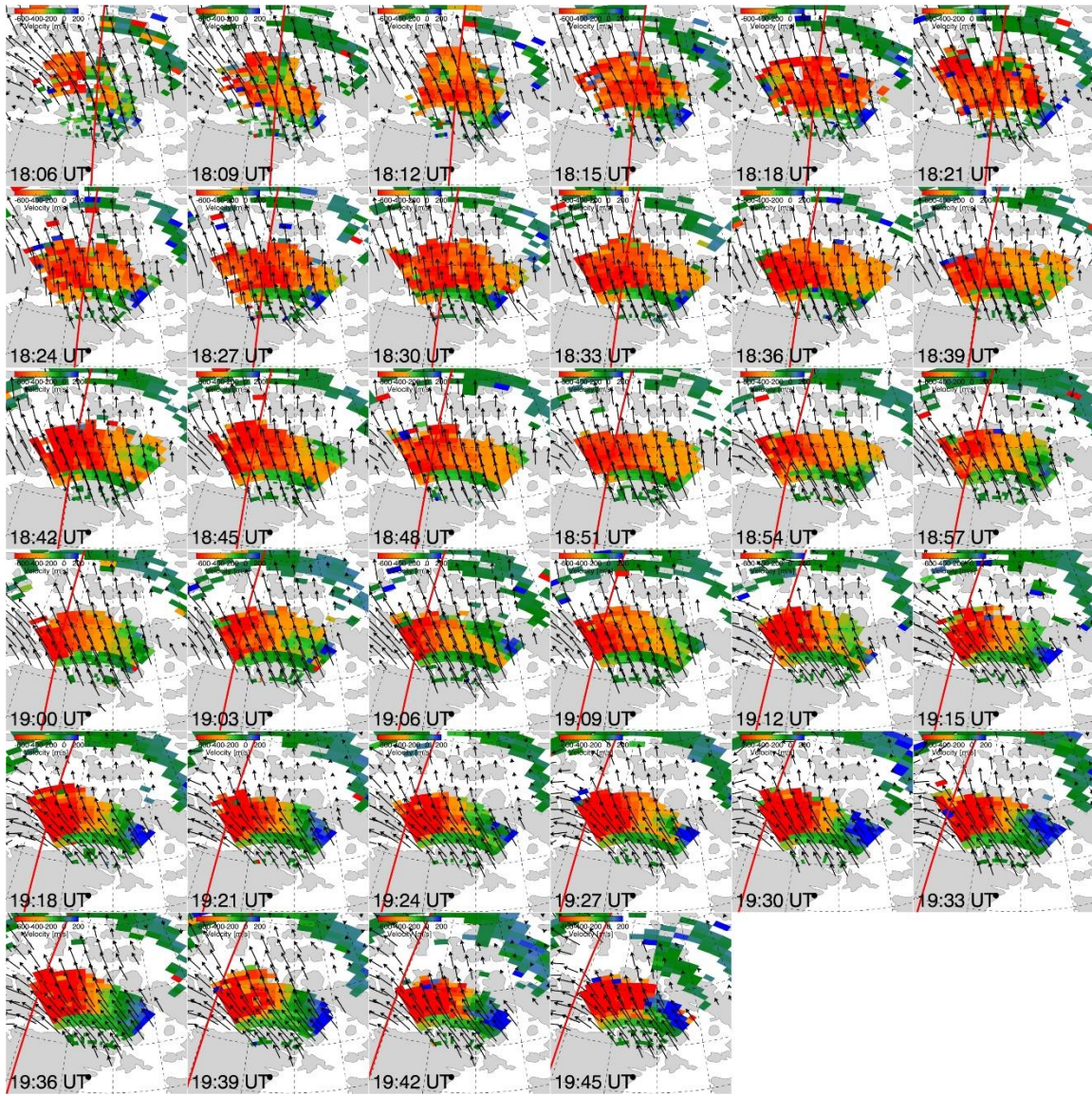

(c) Event 3 (17:15-18:18 UT)

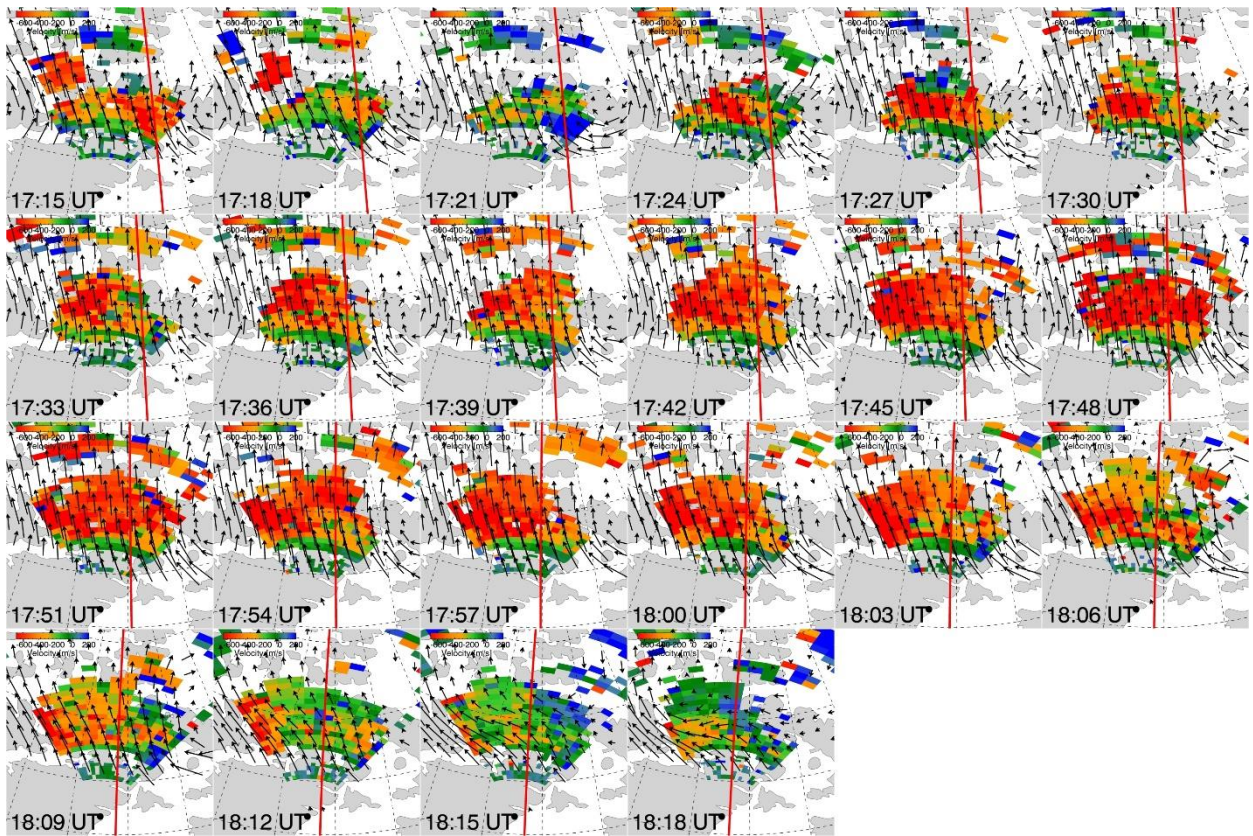

Supplement: Supplementary file 1 — Additional File 1: Figure S1. Line-of-sight plasma velocity within the radar field of view at the time intervals:17:54–19:21 UT during Event 1,18:06–19:45 UT during Event 2, and17:15–18:18 UT during Event 3. Arrows indicate velocity vectors inferred from multiple radars. [file 40623_2024_2101_MOESM1_ESM.pdf]
